# Elastic modulus of recycled aggregate concrete: a multiscale-multiphase modeling approach

**DOI:** 10.1617/s11527-026-03261-8

**Published:** 2026

**Authors:** Osamah H. A. Dehwah, Stephanie S. Watson

**Affiliations:** PREP Associate, Engineering Laboratory, National Institute of Standards and Technology, Gaithersburg, MD 20899, USA; Department of Civil and Systems Engineering, Johns Hopkins University, Baltimore, MD 21218, USA; Engineering Laboratory, National Institute of Standards and Technology, Gaithersburg, MD 20899, USA

**Keywords:** Recycled aggregate concrete, Elastic modulus, Multiscale-multiphase model, Generalized self-consistent, Micromechanics

## Abstract

Concrete is a multiscale, multiphase composite material whose elastic properties are challenging to predict, particularly when recycled aggregates are used. The inclusion of recycled aggregates introduces greater heterogeneity due to residual mortar, microcracks, and variable material properties, all of which complicate modeling efforts. Growing demand for concrete, scarcity of natural aggregates, and the environmental impacts of cement production have increased interest in recycled aggregate concrete (RAC) from construction waste as a sustainable alternative. However, RAC often exhibits reduced mechanical performance due to residual mortar, pre-existing microcracks, and variability in particle properties. While numerous experimental and numerical studies have explored the elastic modulus of RAC, existing theoretical models remain limited. Most simplify concrete as a two-phase system of mortar and aggregate, overlooking the influence of cement paste and hydration products. Poisson’s ratio is commonly neglected, despite natural and recycled aggregates generally exhibiting different values. This study presents a multiphase and multiscale computational framework for predicting the elastic modulus of RAC. The model integrates nanoscale, microscale, and mesoscale interactions using the Mori–Tanaka and Generalized Self-Consistent schemes, incorporating cement paste and clinker hydration products and investigating the influence of differences in Poisson’s ratio between natural and recycled aggregates on the predicted elastic modulus. The framework was validated in two stages and demonstrated good agreement with experimental results, with errors below 10%. A parametric study was performed, showing that the macroscale elastic response is governed primarily by the aggregate volume fraction and elastic stiffness, while the influence of Poisson’ratio is comparatively small.

## 1 Introduction

The growing demand for construction materials has led to a significant increase in the production of concrete, the most widely used construction material worldwide [1, 2]. The multiscale and multiphase nature of concrete complicates the computational prediction of its mechanical behavior. In parallel, the scarcity of natural resources and the environmental impacts of concrete production have driven research into recycled and eco-friendly alternatives in concrete mixtures [3–5]. One promising approach is the use of recycled aggregates derived from demolished concrete structures. Whether reinforced or non-reinforced, such structures generate large volumes of waste, which can be processed and reused as recycled aggregate in new concrete [6]. However, combining recycled and natural aggregates increases the material’s heterogeneity, making it more challenging to develop computational prediction models.

Since aggregates account for 55% to 80% of the total concrete volume [7], the recycling of this material offers significant potential to mitigate resource depletion and landfill disposal [8]. Consequently, recycled aggregates have received growing attention as a sustainable alternative to natural aggregates [9–11]. The recycled aggregates are processed and blended with fresh cement paste to produce recycled aggregate concrete (RAC) [12, 13]. RAC consists of aggregates, residual mortar, commonly referred to as “old mortar”, and may also include supplementary cementitious materials such as fly ash, silica fume, or other additives [14, 15]. In practice, recycled aggregates can be used to replace both coarse and fine aggregates in conventional concrete [8].

In general, RAC is of lower quality than natural concrete for several reasons. The old cement paste adhering to their surfaces leads to higher water absorption capacity, while the crushing of waste concrete reduces stiffness. Furthermore, the weak bond between old and new mortar, along with the cracks generated during the crushing process, significantly reduces the quality of RAC [16–18]. Additionally, the irregular particle shape and surface texture of recycled aggregates contribute to greater variability in the resulting concrete properties [8, 19]. Recycled aggregate typically has a lower specific gravity compared to natural aggregate [8]. The complex interactions between new and old materials in RAC remain an active area of research [20–24], and numerous experimental studies have investigated its mechanical behavior [25–31]. Increasing the replacement ratio of recycled aggregates with natural aggregates has been shown to reduce the elastic modulus, compressive strength, and compressive toughness of concrete specimens [32]. Recycled brick aggregates have also been investigated as substitutes for natural aggregates in concrete and studies have consistently concluded that their use leads to reductions in the concrete performance [5, 32–36].

Although RAC contains pre-existing microcracks, failure tends to localize primarily in the old and new cement paste, as well as in the interfacial transition zones (ITZs), rather than within the aggregate particles themselves [14, 37, 38]. Various constitutive and damage mechanics models have been developed to study recycled aggregate replacement numerically and statistically [3, 12, 18, 39], where Poisson’s ratio was neglected. In parallel, a few theoretical models have been proposed [40, 41], though they focused on two-phase consisting of mortar and aggregates without accounting for the cement paste, as is done in the present study. Sae-Long et al. [14] developed a combined theoretical–numerical model for RAC to study post-peak behavior, but without considering cement paste hydration characteristics or Poisson’s ratio, both of which are considered in the present framework. Investigating the mechanical properties of the different phases is essential, as they play a critical role in evaluating the elastic and overall mechanical behavior of concrete containing recycled aggregates. Natural and recycled aggregates also generally differ in Poisson’s ratio, in addition to elastic modulus; the present study incorporates Poisson’s ratio into the model formulation and investigates its influence on the predicted elastic response.

A theoretical model using multiphase and multiscale approach is presented for predicting the elastic properties of recycled concrete. Moreover, the model integrates the nanoscale, microscale, and mesoscale using micromechanics-based homogenization, specifically the Generalized Self-Consistent (GSC) and Mori–Tanaka schemes. The advantage of this framework lies in evaluating each scale independently and then linking them through composite mechanics, namely homogenization theory. The accuracy of this approach can be further enhanced as more detailed information becomes available on the mechanical properties of the different phases associated with RAC, such as old mortar and ITZ.

Unlike previous models that typically considered two phases, mortar and aggregate, this model incorporates the cement matrix as failure often initiates in the ITZ between mortar and aggregates and mortar composite of sand and cement paste. Including the cement matrix is therefore essential, as it represents the weak layer where cracking is most likely to occur. In this framework, cement, sand, and aggregates are accounted. The cement matrix, comprising clinker phases and hydration products, is modeled using the Mori–Tanaka scheme at the nanoscale and microscale levels. The GSC model is then applied to incorporate sand and aggregates at the mortar and concrete levels (mesoscale). Moreover, an analytical approach based on composite materials theory is developed to evaluate the elastic modulus of RAC. Finally, a parametric study has been conducted to examine how the principal parameters in the model influence the predicted macroscale elastic response.

## 2 Multiphase composite models for concrete

Concrete is a composite material consisting of several distinct phases: coarse aggregate, fine aggregate, and cement paste, which itself contains numerous phases. The properties of these phases strongly influence the effective mechanical behavior of the composite. Over the past decades, numerous composite models have been developed to simplify the internal structure of concrete and to predict its performance. For multiphase composite models, three primary factors govern the effective elastic properties of the material:

1. The elastic properties of each phase (e.g., elastic modulus).
2. The volume fraction of each phase.
3. The orientation and spatial arrangement of the phases. Note that different modeling approaches based on varying assumptions about phase arrangement are to be presented in the following section.

The GSC model uses multiscale and multiphase approaches to describe the internal structure of composite materials and to develop theoretical predictions of their behavior [42]. Originally proposed by Christensen [43] for estimating the elastic properties of composite materials. A fundamental requirement of the model is that the characteristic size of the inhomogeneities, such as aggregate particles, is significantly smaller than the characteristic dimensions of the specimen or representative volume element (RVE). Based on this assumption, a heterogeneous composite can be represented by discrete regions or elements, within which the volume fraction of each phase is considered to be uniform.

To simplify the internal structure, the material is divided into multiple elements, or partitions, as illustrated in Fig. 1a. Each element is represented by a spherical inclusion, Fig. 1b, with radius $r_{1}$ embedded in a concentric matrix sphere with radius $r_{2}$ and this composite unit is then embedded in an effective medium, Fig. 1c. It is always assumed that the ratio r1r2 remains constant across all elements.

A key requirement of this modeling approach is a broad particle size distribution, which ensures that smaller particles fill the voids between larger ones. This condition reflects the case of concrete, where particle sizes of cement, fine aggregate and coarse aggregate span from micrometers to decimeters, enabling efficient packing across multiple scales. For this reason, the GSC model is employed in this study to evaluate the elastic modulus of recycled concrete. At each scale, the effective elastic modulus is calculated using the following expression:

(1) $$E_{\text{eff}}=\frac{9k_{\text{eff}}G_{\text{eff}}}{3k_{\text{eff}}+G_{\text{eff}}}$$

where $k_{\text{eff}}$ and $G_{\text{eff}}$ represent the effective bulk modulus and effective shear modulus, respectively, with their formulations given as follows:

(2) $$k_{\text{eff}}=k_{\text{m}}+\frac{c_{i}\left(k_{i}-k_{\text{m}}\right)}{1+\left(1-c_{i}\right)\frac{\left(k_{i}-k_{\text{m}}\right)}{k_{\text{m}}+4/3G_{\text{m}}}}$$

(3) C1(GeffGm)2+2C2(GeffGm)+C3=0

Subscripts i and m refer to the inclusion and matrix phases, respectively. Here, is the bulk modulus, G is the shear modulus, and c represents the volume fraction. The dimensionless coefficients, and can be obtained following the formulations in Appendix A, which were derived in [44].

### 2.1 Other schemes for evaluating the elastic modulus

Several classical two-phase models are considered here as theoretical reference formulations for comparison with the GSC model. These models employ different idealized assumptions regarding phase arrangement and are not all intended to reproduce the actual microstructure of concrete. The parallel (Voigt) and series (Reuss) schemes represent limiting phase arrangements, while the Hashin–Shtrikman model provides more restrictive bounds. The Counto and Hirsch models provide additional analytical estimates based on different assumptions regarding the interaction and arrangement of the constituent phases [45]. Collectively, these models provide theoretical bounds or analytical estimates for the effective elastic modulus, while the GSC model is employed here as the predictive homogenization scheme. To illustrate their differences, Fig. 2 presents the schematic representation of a two-phase system, where phase 1 denotes the inclusion and phase 2 the matrix—corresponding, in the case of concrete, to aggregate and mortar, respectively.

In the parallel scheme, the two phases are aligned with the loading direction, Fig. 2a, which produces the highest upper bound. In contrast, the series model assumes that the phases are arranged perpendicular to the loading direction, Fig. 2b, resulting in the lowest bound [46–49]. The Hashin–Shtrikman (H–S) model [50–52], derived from variational principles in the linear theory of elasticity and effective magnetic permeability of composites, provides both an upper bound (Fig. 2c) and a lower bound (Fig. 2d); the lower bound is excluded from the present comparison, as it corresponds to an arrangement in which aggregate surrounds the mortar, contrary to the actual microstructure of concrete. The Counto model [53] combines features of the parallel and series schemes, where the matrix orientation differs but inclusions remain centered. This leads to two variations: Counto-I (Fig. 2e) and Counto-II (Fig. 2f). Lastly, the Hirsch model [54] represents an average of the parallel and series schemes (Fig. 2g) and yields results very close to those of Counto-I. Table 1 summarizes the effective elastic modulus formulations for these models, where the effective modulus of concrete is expressed in terms of two phases: inclusion $\left(i\right)$ and matrix $\left(m\right)$.

The phase arrangements of the models are illustrated in the schematic diagrams in Fig. 2. The elastic moduli of the inclusions and matrix were assumed to be 60 GPa and 30 GPa, respectively. To compare their predictive performance, the normalized elastic modulus $\left(E_{\text{eff}}/E_{i}\right)$ is plotted against the inclusion volume fraction $\left(c_{\text{i}}\right)$ in Fig. 3. These models provide reasonable estimates at low inclusion volume fractions; however, at higher fractions their predictions deviate significantly, and as mentioned earlier the coarse aggregate volume fractions would range from 55 to 80%. Since the elastic modulus is expressed in the order of 10^9^ (Pa), even small discrepancies in the volume fraction can lead to magnified stress differences. It is worth noting that the GSC model consistently lies between these bounds. Since natural and recycled aggregates differ in both elastic modulus and Poisson’s ratio, the GSC model accounts for both properties in the homogenization process.

The GSC model is applied to predict the elastic modulus of concrete containing both natural and recycled aggregates. Numerous experimental studies have investigated the elastic properties of concrete made with natural aggregates, recycled aggregates, or a combination of both, where recycled aggregates are used as a replacement for natural ones [8, 32, 36, 40, 55]. In this study, the GSC model is employed in two stages:

- *Stage I*: The effective elastic modulus is first evaluated for concrete made entirely with natural aggregates, and separately for concrete made entirely with recycled aggregates.
- *Stage II*: The elastic modulus of concrete containing both natural and recycled aggregates, coexisting within the same mix, is then predicted using the GSC model.

### 2.2 Stage I: elastic modulus for natural aggregate concrete (NAC) and RAC

In this work, three distinct phases are considered: aggregate, sand, and cement paste. Using the GSC scheme, the model is divided into two subsystems, each involving two phases at a time, as illustrated in Fig. 4a and b. In the first subsystem, sand is treated as the inclusion and cement paste as the matrix, together forming the new mortar. In the second subsystem, this mortar (the effective medium obtained from the first subsystem) is treated as the matrix, while aggregate is considered as the inclusion; together, these constitute the effective medium representing concrete. For clarity, new mortar refers to the mortar formed by the cement paste and sand of the new concrete, whereas old mortar refers to the residual mortar adhered to the recycled aggregate. The present study focuses on the replacement of coarse aggregate, with natural sand considered as the fine aggregate. For concrete containing recycled fine aggregate, the proposed framework could be extended by introducing an additional phase at the mortar level to represent the recycled fine aggregate, resulting in a four-phase model.

### 2.3 Mori–Tanaka model

The elastic modulus of cement paste is evaluated using the Mori–Tanaka scheme. Cement paste contains a variety of hydration products and crystals that are randomly distributed, making it quite different from the other phases at higher scales e.g., coarse and fine aggregates. In addition, certain phases, such as ettringite, exhibit non-spherical shapes. Therefore, the Mori–Tanaka scheme is best suited for modeling cement paste, as it can account for these microstructural characteristics and heterogeneities [56]. The stiffness tensor of a designated component is given by the following expression [57]:

(11) $$C_{\text{comp}}=\sum_{\text{p}={\text{p}}_{1},{\text{p}}_{2},M}f_{\text{p}}C_{\text{p}}\;:\;A_{\text{p}}$$

here, $f_{\text{p}}$ represents the volume fraction of phase $p;C_{\text{p}}$ is the fourth-order stiffness tensor of phase p; and $A_{\text{p}}$ is the fourth-order strain concentration tensor of phase p, which accounts for the shapes of the constituent phases and depends on the Eshelby tensor, and given by Eq. (12). Further elaboration on Eq. (11) is given in Eq. (13), where θ and ϕ are the Euler angles which describe the orientation of the rigid body with respect to a fixed coordinate system.

(12) $$A_{\text{p}}={\left[I+S_{\text{p}}\;:\;\left(C_{\text{M}}^{-1}\;:\;C_{\text{p}}-I\right)\right]}^{-1}\;:\;{\left\{\sum_{\text{p}=\text{P}1,\text{P}2,\text{P}3}f_{\text{p}}{\left[I+S_{\text{p}}\;:\;\left(C_{\text{M}}^{-1}\;:\;C_{\text{p}}-I\right)\right]}^{-1}\right\}}^{-1}$$

(13) $$C_{\text{comp}}=\left\{\int_{\theta =0}^{\pi }\int_{\phi =0}^{2\pi }f_{\text{p}3}C_{\text{p}3}\;:\;{\left[I+S_{\text{p}3}\left(\theta ,\phi \right)\;:\;\left(C_{\text{M}}^{-1}\;:\;C_{\text{p}3}-I\right)\right]}^{-1}\frac{\text{sin}\left(\theta \right)}{4\pi }d\theta d\phi +\sum_{\text{p}=\text{P}1,\text{P}2}f_{\text{p}}C_{\text{p}}\;:\;{\left[I+S_{\text{p}}\;:\;(C_{\text{M}}^{-1}\;:\;C_{\text{p}}-I\right]}^{-1}+f_{\text{M}}C_{\text{M}}\right\}\;:\;{\left\{\int_{\theta =0}^{\pi }\int_{\phi =0}^{2\pi }f_{\text{p}3}\;:\;{\left[I+S_{\text{p}3}\left(\theta ,\phi \right)\;:\;\left(C_{\text{M}}^{-1}\;:\;C_{\text{p}1}-I\right)\right]}^{-1}\frac{\text{sin}\left(\theta \right)}{4\pi }d\theta d\phi +\sum_{\text{p}=\text{P}1,\text{P}2}f_{\text{p}}\;:\;{\left[I+S_{\text{p}}\;:\;(C_{\text{M}}^{-1}\;:\;C_{\text{p}}-I\right]}^{-1}+f_{\text{M}}I\right\}}^{-1}$$

where I denotes the fourth-order identity tensor, and $S_{\text{p}}$ is the fourth-order Eshelby tensor associated with phase p. Herein, an equivalent homogeneous medium is considered, consisting of a matrix phase (M) and multiple inclusion phases with different shapes and orientations $\left(P_{1},P_{2},P_{3}\right)$, as illustrated in Fig. 5 and discussed in the following section.

The hardened cement paste is a multiphase composite made up of clinker, calcium hydroxide (CH), aluminates, and capillary pores, all embedded within a calcium silicate hydrate (C–S–H) gel matrix. The C–S–H phase can be further differentiated into three categories: a) Low-density (LD) C–S–H; b) High-density (HD) C–S–H; c) Ultrahigh-density (UHD) C–S–H [58, 59]. In this framework, clinker particles are concentrated and surrounded by UHD C–S–H, which in turn is enveloped by HD C–S–H, see Fig. 5(a). LD C–S–H is considered the matrix of the cement paste. Accordingly, the system can be treated as a multiscale scheme where the clinker and UHD C–S–H together form the sub-inclusion, while this combined inclusion with HD C–S–H constitutes the equivalent inclusion.

Moreover, the theoretical morphology of each component of hardened cement paste is illustrated in Fig. 5. Clinker is modeled as half of a prolate spheroid and half of an oblate spheroid, with an aspect ratio of 0.81 [60]. The UHD and HD C–S–H phases are assumed to be oriented parallel to the clinker and therefore share the same aspect ratio. Calcium hydroxide is modeled as a prolate spheroid with an aspect ratio of 0.70. Ettringite (aluminate), which exhibits an intrinsic needle-like morphology, is also represented as a prolate spheroid with an aspect ratio of 0.69 [61]. Voids are assumed to be spherical. Additional details on aspect ratios and shape factors can be found in [60, 62].

The theoretical model for clinker takes into account the cement type and water-to-cement ratio. Clinker is composed of four principal phases: (1) Alite (C_3_S), (2) Belite (C_2_S), (3) Tricalcium aluminate (C_3_A), and (4) Ferrite (C_4_AF). It is therefore more reasonable to treat clinker as a composite material consisting of these four phases rather than as an isotropic and homogeneous solid. Based on the morphology described in [63] and modeled using the Mori–Tanaka scheme, the theoretical clinker model is illustrated in Fig. 5(b). In this representation, C_3_S is considered the matrix, while the remaining phases act as inclusions, as shown in Eq. (14). This representation is used to obtain the effective elastic properties of clinker for subsequent homogenization at the cementpaste, mortar, and concrete scales.


(14) $$C_{\text{clinker}}=\left\{\sum_{\text{p}={\text{C}}_{2}\text{S},{\text{C}}_{4}\text{AF},{\text{C}}_{3}\text{A}}f_{\text{p}}^{\text{clinker}}C_{\text{p}}\;:\;{\left[I+S_{\text{p}}\;:\;\left(C_{{\text{C}}_{3}\text{S}}^{-1}\;:\;C_{\text{p}}-I\right)\right]}^{-1}+f_{{\text{C}}_{3}\text{S}}^{\text{clinker}}C_{{\text{C}}_{3}\text{S}}\right\}\;:\;{\left\{\sum_{\text{p}={\text{C}}_{2}\text{S},{\text{C}}_{4}\text{AF},{\text{C}}_{3}\text{A}}f_{\text{p}}^{\text{clinker}}{\left[I+S_{\text{p}}\;:\;\left(C_{{\text{C}}_{3}\text{S}}^{-1}\;:\;C_{\text{p}}-I\right)\right]}^{-1}+f_{{\text{C}}_{3\text{S}}}^{\text{clinker}}I\right\}}^{-1}$$

### 2.4 Phase volume fractions for multiscale homogenization

The volume fractions of the constituent phases are required to determine the elastic properties at each hierarchical level. The total volume of cement paste $\left(V_{\text{cp}}\right)$ is defined as the sum of the volumes of unreacted clinker (core), capillary pores (capillary), and hydration products such as CSH and CH, hydrated calcium aluminates phases (AFm), ettringite (Aft). This relationship is expressed as follows:

(15) $$V_{\text{cp}}=V_{\text{core}}+V_{\text{capillary}}+V_{\text{CSH}}+V_{\text{CH}}+V_{\text{AFm}}+V_{\text{AFt}}$$

The volume fractions of these phases are defined by Xi and Jennings [64] as follows:

(16) $$f_{\text{core}}=C\left[\frac{\left(1-\alpha \right)}{\rho_{\text{c}}}\right]P$$

(17) $$f_{\text{capillary}}=[(1-C)-C\Omega ]\text{P}$$

(18) $$C=\frac{1}{1+w/c}$$

(19) $$P=\frac{1+w/c}{1+w/c\left(\rho_{c}/\rho_{w}\right)}\rho_{c}$$

(20) $$\Omega =0.437\alpha_{{\text{C}}_{3}\text{S}}W_{{\text{C}}_{3}\text{S}}+0.503\alpha_{{\text{C}}_{2}\text{S}}W_{{\text{C}}_{2}\text{S}}+0.397\alpha_{{\text{C}}_{3}\text{A}}W_{{\text{C}}_{3}\text{A}}+0.136\alpha_{{\text{C}}_{4}\text{AF}}W_{{\text{C}}_{4}\text{AF}}$$

(21) $$f_{\text{CSH}}=f_{\text{gel}\;\text{pores}}+f_{\text{CSH}\;\text{solid}}$$

(22) $$f_{\text{CSH}\;\text{solid}}=C\left(0.278\alpha_{{\text{C}}_{3}\text{S}}W_{{\text{C}}_{3}\text{S}}+0.369\alpha_{{\text{C}}_{2}\text{S}}W_{{\text{C}}_{2}\text{S}}\right)P$$

(23) $$f_{\text{gel}\;\text{pores}}=0.219f_{\text{CSH}\;\text{solid}}$$

(24) $$f_{\text{CH}}=C\left(0.189\alpha_{{\text{C}}_{3}\text{S}}W_{{\text{C}}_{3}\text{S}}+0.058\alpha_{{\text{C}}_{2}\text{S}}W_{{\text{C}}_{2}\text{S}}\right)P$$

(25) $$f_{\text{AFm}}=C\left(0.849\alpha_{{\text{C}}_{3}\text{A}}W_{{\text{C}}_{3}\text{A}}+0.472\alpha_{{\text{C}}_{4}\text{AF}}W_{{\text{C}}_{4}\text{AF}}\right)P$$

(26) $$f_{\text{AFt}}=1-\left(f_{\text{core}}+f_{\text{CH}}+f_{\text{AFm}}+f_{\text{capillary}}+f_{\text{CSH}}\right)$$

where $\rho_{\text{c}}$ and $\rho_{\text{w}}$ denote the densities of cement and water, respectively, expressed in g/cm^3^, with a density ratio of $\rho_{\text{c}}/\rho_{\text{w}}=3.15$. The volume fractions of the remaining constituent phases are defined in Appendix B. The degree of hydration $\left(\alpha \right)$ is approximated using the Avrami equation, given in Eq. (27). The overall degree of hydration is computed as a weighted average of the individual clinker phase contributions, as detailed by Taylor [65]. Here, $W_{i}$ represents the initial mass fraction of each reacting clinker phase $\left(i={\text{C}}_{3}\text{S},{\text{C}}_{2}\text{S},{\text{C}}_{3}\text{A},{\text{C}}_{4}\text{AF}\right)$.

(27) $$\alpha_{i}=1-\text{exp}\left[-a_{i}{\left(t-b_{i}\right)}^{c_{i}}\right]$$

where t represents time (days); the coefficients $a_{i},b_{i}$, and $c_{i}$ are listed in Table 2, together with the corresponding elastic moduli and Poisson’s ratios of the constituent phases. The overall degree of hydration of the cement-based material is then evaluated as follows:

(28) $$\alpha =\frac{\sum_{i}\alpha_{i}W_{i}}{\sum_{i}W_{i}}$$

The volume fractions of gravel, sand, cement paste, and water are determined from the mix design parameters as follows.

1- At the mortar scale, the volume fractions of sand $\left(f_{\text{s}}\right)$ and cement paste $\left(f_{\text{cp}}\right)$ are computed as:

(29) $$f_{\text{s}}=V_{\text{s}}/\left(V_{\text{s}}+V_{\text{c}}+V_{\text{w}}\right)$$

(30) $$f_{\text{cp}}=1-f_{\text{s}}$$

where $V_{\text{s}},V_{\text{c}}$, and $V_{\text{w}}$ denote the volumes of sand, cement, and water in the concrete mixture, respectively, expressed in cm^3^. The volume of a generic phase $p\left(V_{\text{p}}\right)$ is computed as the ratio of its mass to its density, i.e., $V_{\text{p}}=W_{\text{p}}/\rho_{\text{p}}$, where $W_{\text{p}}$ is given in grams and $\rho_{\text{p}}$ in g/cm^3^.

2- At the concrete scale, the volume fractions of gravel $\left(f_{\text{g}}\right)$ and mortar $\left(f_{\text{m}}\right)$ are computed as:

(31) $$f_{\text{g}}=V_{\text{g}}/\left(V_{\text{s}}+V_{\text{c}}+V_{\text{w}}+V_{\text{g}}\right)$$

(32) $$f_{\text{m}}=1-f_{\text{g}}$$

where $V_{\text{g}}$ is the volume of gravel in concrete given in $cm^{3}$.

In this framework, natural aggregate is modeled solely as the inclusion, as shown in Fig. 6a, while recycled aggregate is modeled solely as the inclusion, as shown in Fig. 6b. It should be noted that recycled aggregate typically contains adhered old mortar and ITZ, as illustrated in Fig. 6c. In the present framework, recycled aggregate is represented as an effective medium, such that the collective influence of its heterogeneous constituents, including adhered old mortar and ITZ, is reflected in its overall effective elastic behavior rather than modeled as separate phases. Because reliable experimental data on the elastic properties of old mortar and ITZ are limited, these phases are not modeled separately but could be incorporated as more data become available.

In summary:

- *Scale 1 (Concrete)*: At this largest scale, the effective elastic modulus of concrete is evaluated. The inclusions are the coarse aggregates, while the surrounding mortar (sand + cement paste) is treated as the matrix.
- *Scale 2 (Mortar)*: At this intermediate scale, sand particles are modeled as inclusions embedded in a cement paste (CP) matrix.
- *Scale 3 (Cement Paste)*: The effective elastic modulus of cement paste is determined using the Mori–Tanaka scheme, where the different hydration products, clinker phases, and voids are taken into considered.

A flowchart illustrating the overall workflow is shown in Fig. 7.

### 2.5 Stage II: elastic modulus for concrete

At this stage, the GSC model focuses on mixtures of natural and recycled aggregates. Since the elastic modulus of RAC is lower and consists of heterogeneous phases (e.g., old mortar and old ITZ), it is more appropriate to treat RAC as the matrix, with NAC serving as the inclusion. The elastic moduli of both RAC and NAC were evaluated in the previous stage. Accordingly, the GSC scheme is represented in Fig. 8. The results and implications of the developed model are presented in the next section.

## 3 Results and discussions

### 3.1 Stage I validation

To validate the first stage of the developed model, experimental data on concrete containing either natural aggregates (NA) or recycled aggregates (RA) were considered. Several relevant experimental studies are available in the literature. Table 3 summarizes the experimental datasets, including the sample labels as in the references, mix proportions, densities, and Poisson’s ratios. The values were obtained from the references or assumed where not reported. For the three-phase model described earlier, the cement and moisture contents, material densities, and Portland cement type are required as inputs to the Mori–Tanaka scheme. Unless otherwise specified in the references, Type I cement with a density of 3.14 g/cm^3^ was assumed for the theoretical model. The chemical compositions of the main components required for the Mori–Tanaka scheme are given in Table 4. For the GSC model, the fine aggregate (FA) and coarse aggregate (CA) contents, along with their densities and elastic properties, were also required. For the theoretical validation, the elastic modulus was evaluated at 28 days for all datasets, except for Nassar and Soroushian [66], for which it was evaluated at 14 days. A comparison between the experimental results and the GSC predictions is presented in Table 3. The maximum error across all samples was found to be less than 10%, demonstrating good agreement between experimental and theoretical values. However, it is noteworthy that RAC mixes (indicated by “*” in Table 3) exhibited slightly higher prediction errors than mixes containing natural aggregates. This discrepancy arises from the inherent heterogeneity of RAC, including the presence of adhered old mortar, the ITZ, increased water absorption, and microcracks. Finally, it should be noted that the proposed framework simplifies the complex microstructure by combining the different sub-phases into effective phases, as schematically illustrated previously in Fig. 6

### 3.2 Stage II validation

The second stage of the GSC model relies primarily on the elastic moduli of natural aggregates concrete $\left(E_{\text{NCA}}\right)$ and recycled aggregates concrete $\left(E_{\text{RCA}}\right)$, which were validated in the previous section. Conveniently, the same experimental datasets can also be used to validate this stage, where a range of replacement ratios are examined. Zheng et al. [32] and Ji and Wang [36] studied five levels (0%, 25%, 50%, 75%, and 100%), Yan et al. [40] tested ten levels in 10% increments from 0 to 100%, Liu et al. [55] used six levels (0%, 20%, 40%, 60%, 80%, and 100%), and Yang et al. [8] investigated four levels (0%, 30%, 50%, and 100%). As described earlier, in the GSC framework $E_{\text{NCA}}$ is treated as the inclusion and $E_{\text{RCA}}$ as the matrix. The predicted results are compared with experimental data in Fig. 9. Overall, the theoretical GSC model shows good agreement with experiments across the different replacement ratios of natural coarse aggregate with recycled coarse aggregate. To provide further insight, the error between experimental and theoretical predictions is plotted in Fig. 10.

## 4 Parametric study

A parametric study was conducted to examine the macroscale response of the developed model to variations in the principal parameters governing the NAC–RAC system: the stiffness ratio $\left(E_{\text{NAC}}/E_{\text{RAC}}\right)$, the RAC volume fraction, and the ratio of the Poisson’s ratios $\left(v_{\text{NAC}}/v_{\text{RAC}}\right)$. Figure 11a shows that increasing the RAC volume fraction reduces $E_{\text{eff}}$, with a more pronounced reduction at higher stiffness ratios. At 0% RAC volume fraction, the inclusion phase consists entirely of natural aggregate concrete, whereas at 100% RAC volume fraction it consists entirely of recycled aggregate concrete. Furthermore, Fig. 11b shows that, for a given stiffness ratio, a lower RAC replacement produces a higher effective elastic modulus. Both trends are physically consistent with the lower stiffness of RAC relative to NAC, which is generally associated with adhered old mortar, higher porosity, and microcracking. Consequently, a higher RAC fraction increases the proportion of the lower-stiffness phase, thereby reducing the overall elastic stiffness of the composite.

The influence of Poisson’s ratio is shown in Fig. 11c, where $v_{\text{NAC}}/v_{\text{RAC}}$ was varied from 1 to 2 at a fixed stiffness ratio $\left(E_{\text{NAC}}/E_{\text{RAC}}=3\right)$. The resulting change in $E_{\text{eff}}$ is small, with a maximum change of approximately 0.35% across the RAC volume fractions considered. This indicates that, although the Poisson’s ratios of natural and recycled aggregates may differ, their influence on the predicted effective elastic modulus is relatively small within the investigated range.

## 5 Conclusions

This study developed a multiscale, multiphase theoretical framework based on the generalized self-consistent model and Mori–Tanaka scheme to evaluate the elastic modulus of RAC. The main conclusions are summarized as follows:

1. Most existing models treat concrete as a two-phase system (mortar and aggregate) and neglect the cement paste phase. Natural and recycled aggregates generally exhibit different Poisson’s ratios, an effect commonly neglected in existing formulations. The present framework incorporates cement paste as a distinct phase and investigates the influence of this Poisson’s ratio difference on the predicted elastic modulus.
2. The Mori–Tanaka approach was used to account for the heterogeneity and shape orientation of cement hydration products and clinker phases, resulting in a more representative multiscale model of the cementitious matrix.
3. The GSC model was developed and validated in two stages:
  - Stage I: The elastic moduli of NAC and RAC were evaluated separately, achieving errors of less than 10% compared with experimental data.
  - Stage II: The full model, in which natural and recycled aggregates coexist, was validated against experimental data, showing good agreement with errors below 10%.
4. The parametric study showed that the macroscale elastic response is primarily influenced by the RAC volume fraction and the stiffness ratio $E_{\text{NAC}}/E_{\text{RAC}}$. Increasing the RAC fraction reduces the effective elastic modulus, with a more pronounced reduction at higher stiffness ratios. Although natural and recycled aggregates may have different Poisson’s ratios, varying $\nu_{\text{NAC}}/\nu_{\text{RAC}}$ over the investigated range produced a relatively small change in the predicted effective elastic modulus.
5. The proposed framework provides a basis for further refinement as more experimental data become available on the ITZ and old mortar. Incorporating these phases could further improve the prediction accuracy of the model.

## Figures and Tables

**Fig. 1:**
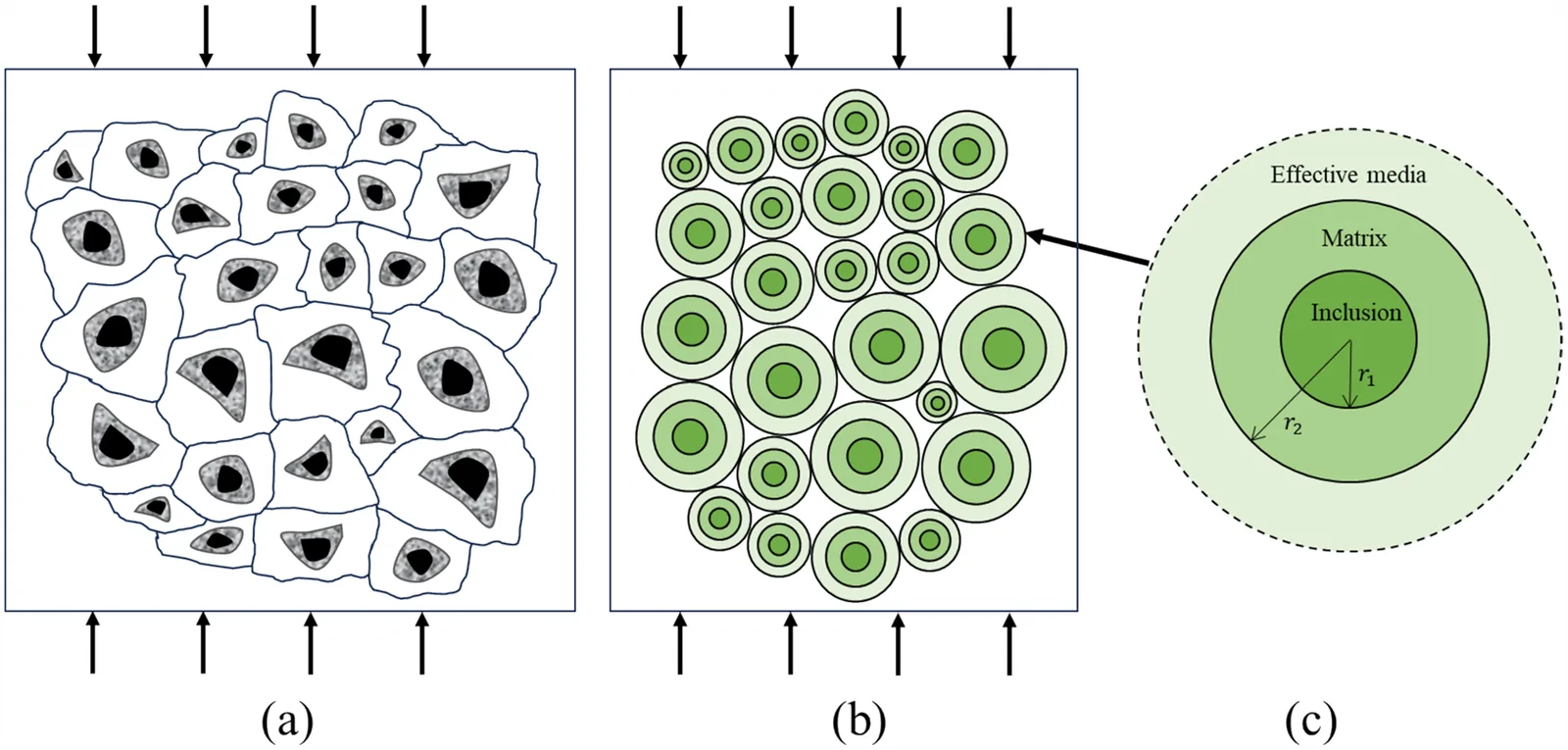
**a** Partitioning multi-phase composite into different elements; **b** simplified internal structure (spherical elements); **c** representative basic unit of the GSC model

**Fig. 2:**
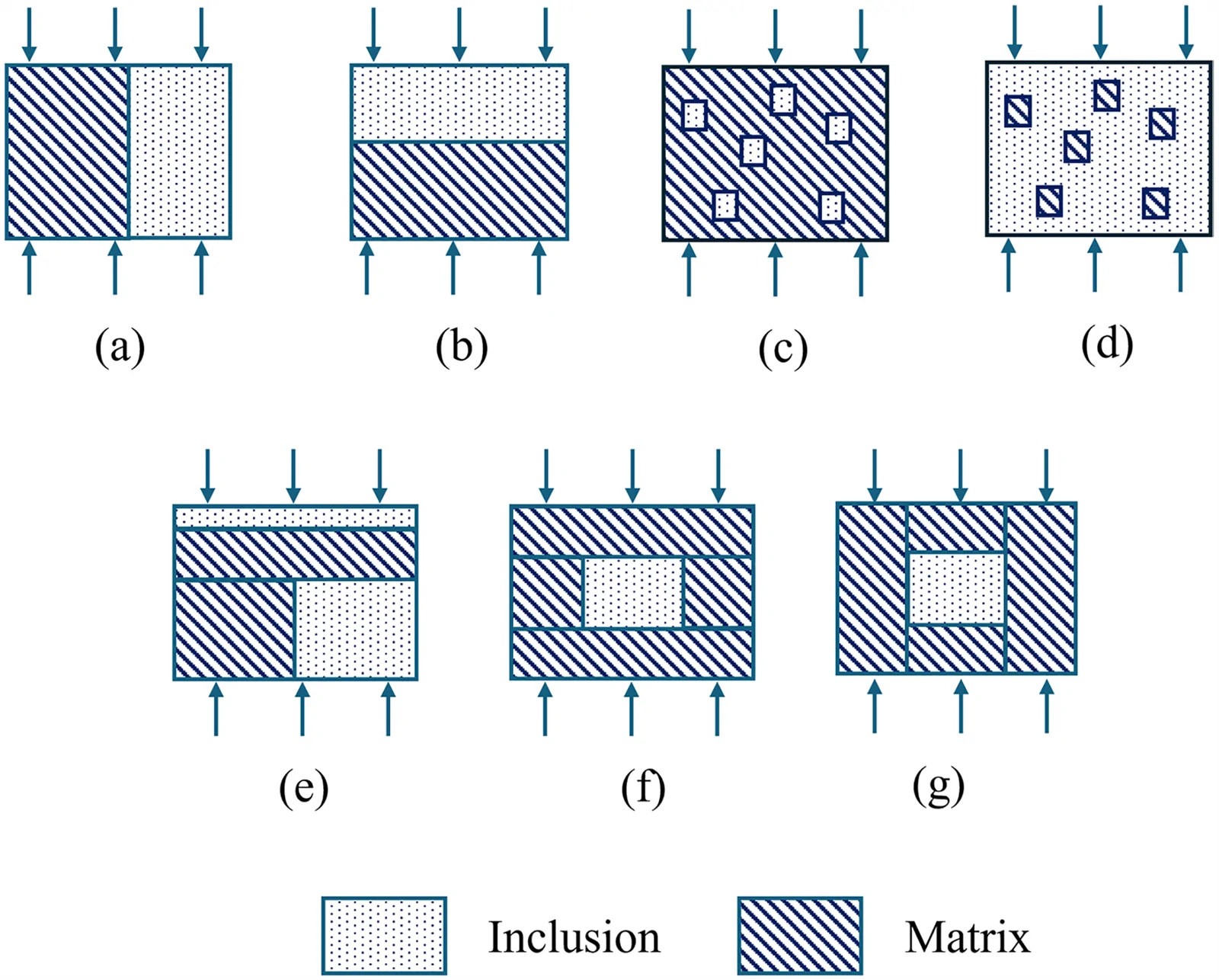
Schematic of a two-phase system with different composite models **a** Parallel; **b** Series; **c** H–S upper bound; **d** H–S lower bound; **e** Counto-I; **f** Counto-II; **g** Hirsch

**Fig. 3:**
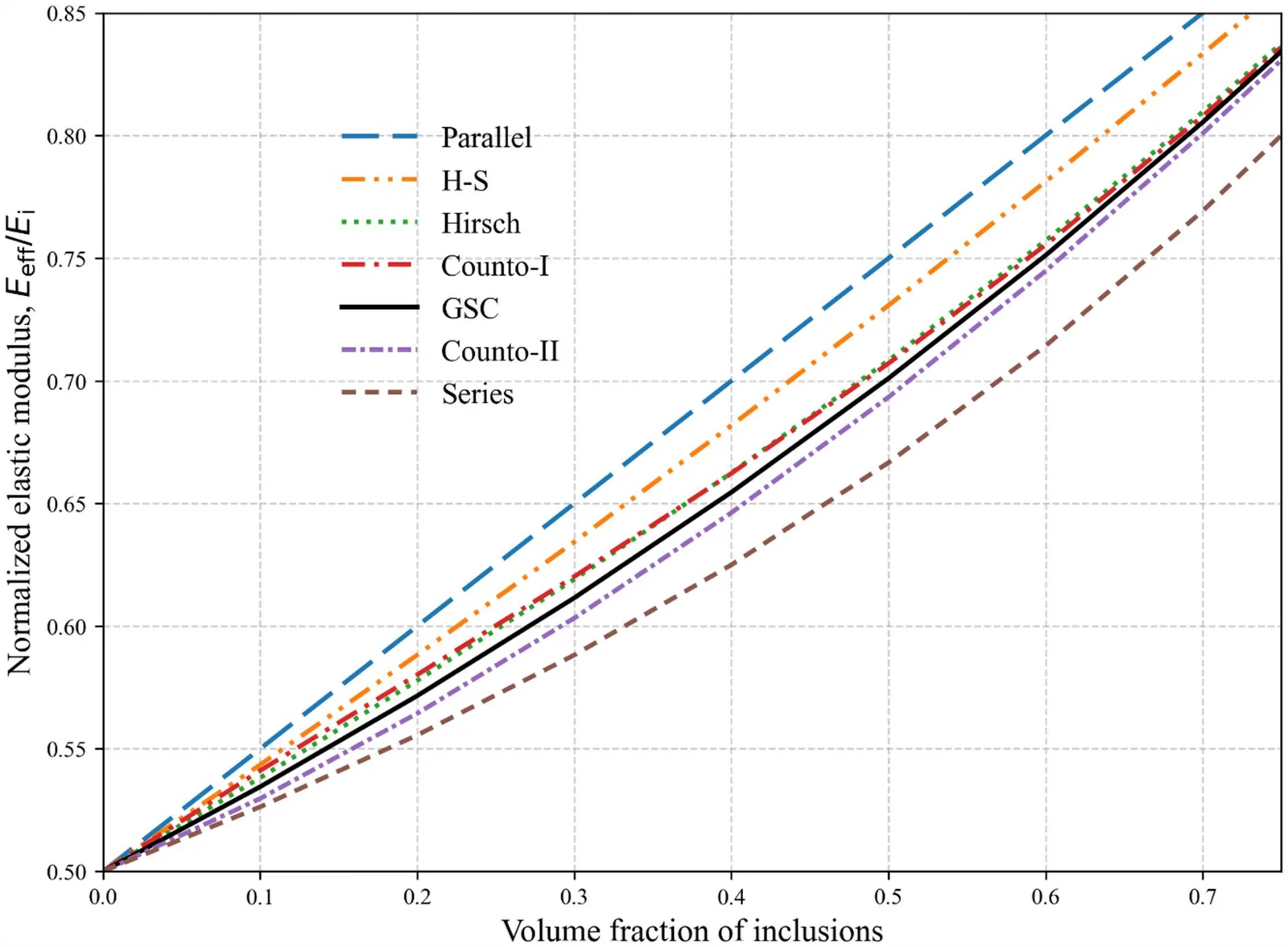
Elastic modulus of two phase composites

**Fig. 4:**
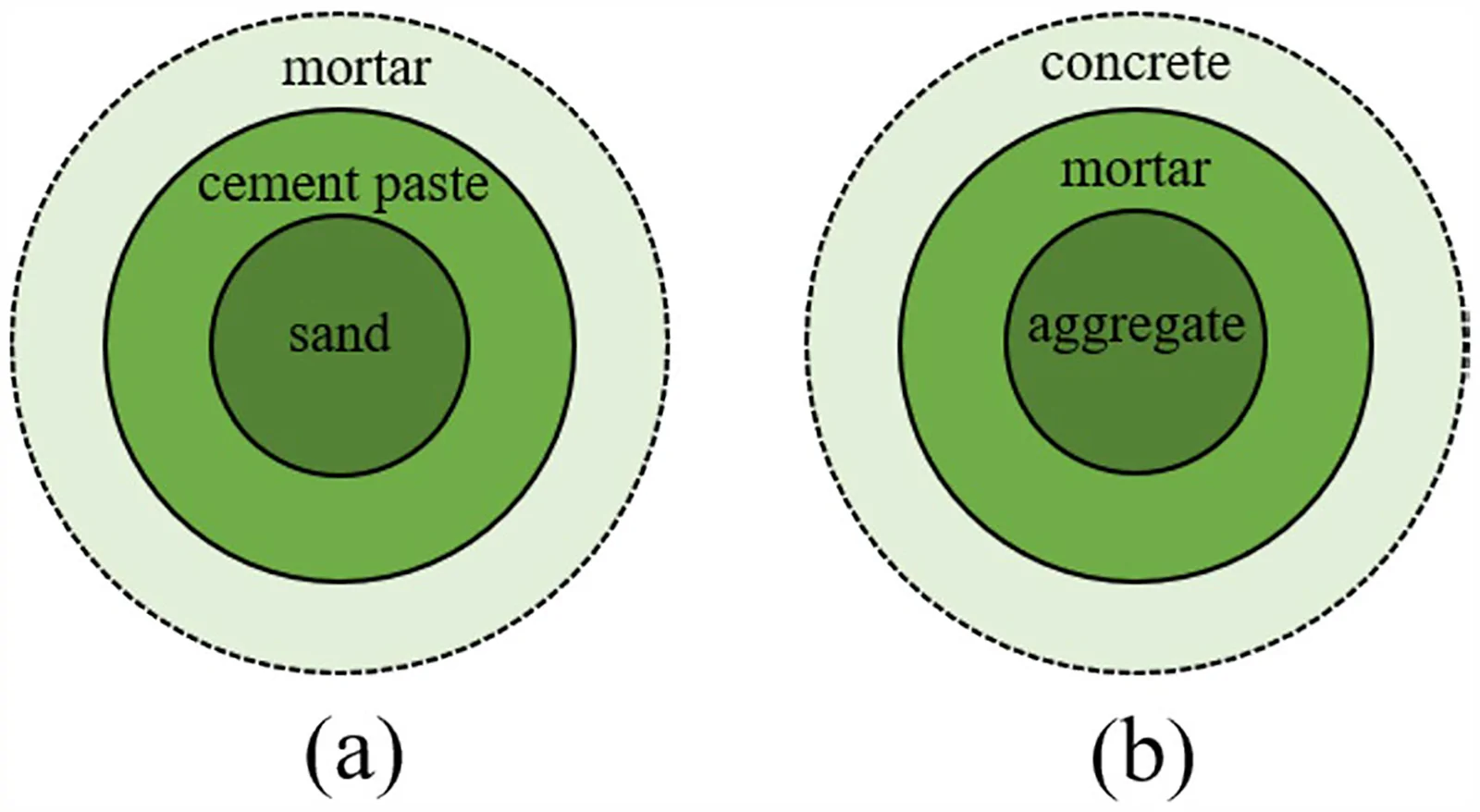
Three-phase model illustration using the GSC schemes

**Fig. 5:**
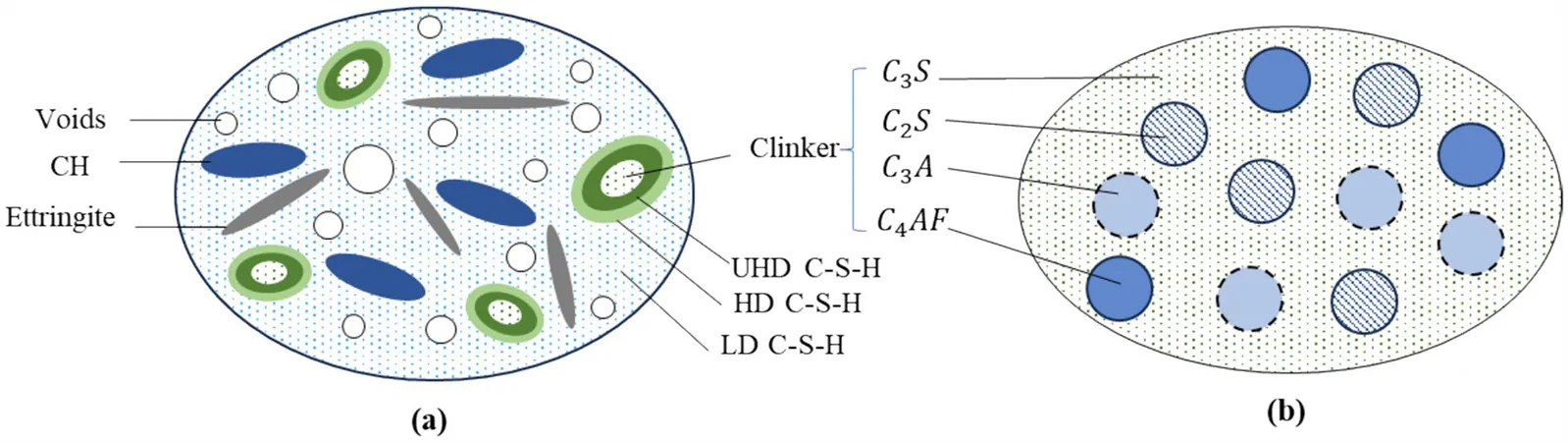
Theoretical model for cement paste **a** inclusion morphology **b** clinker

**Fig. 6:**
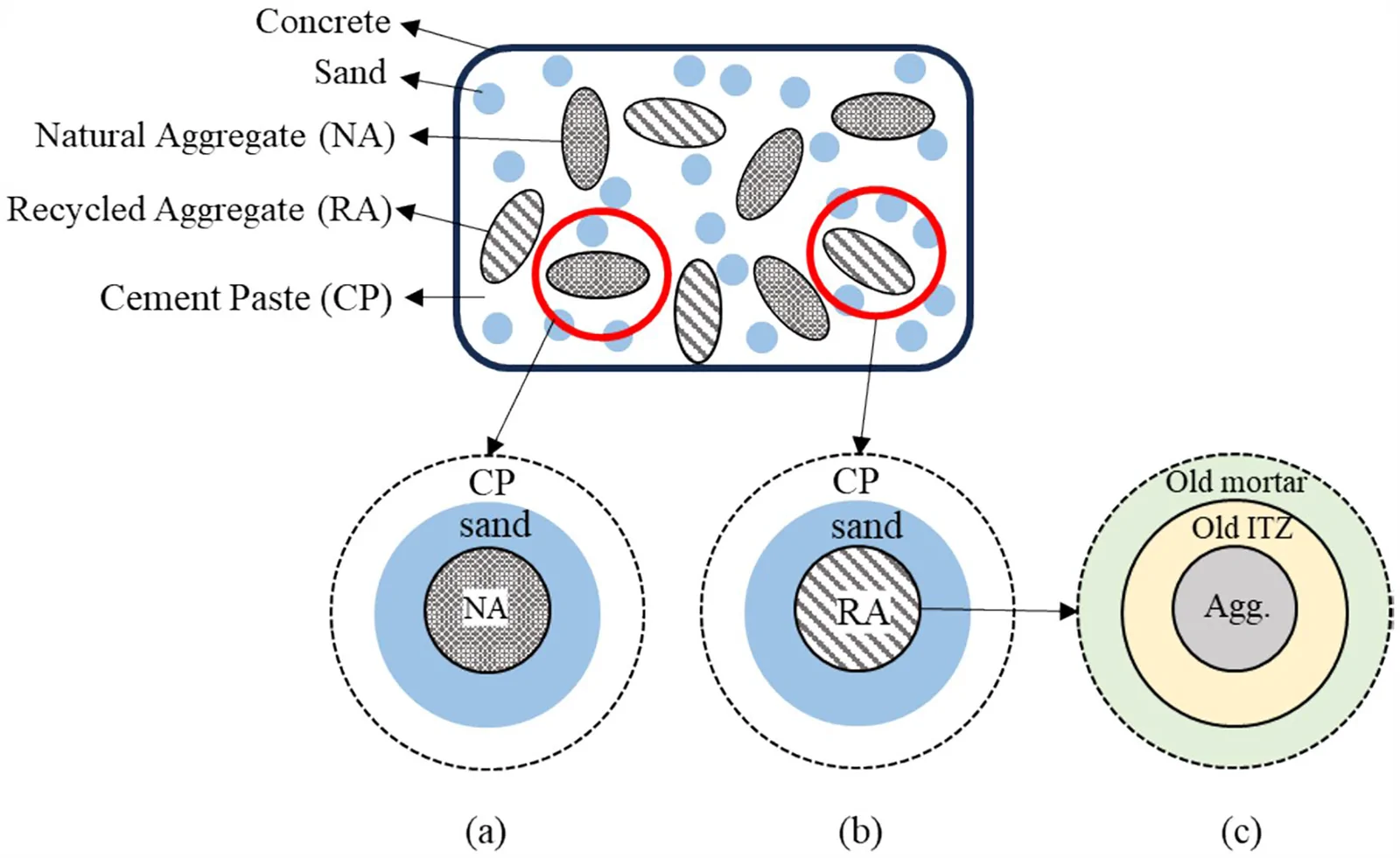
Schematic diagram of the GSC representation for **a** NAC, **b** RAC, and **c** conceptual representation of recycled aggregate with ITZ and old mortar

**Fig. 7:**
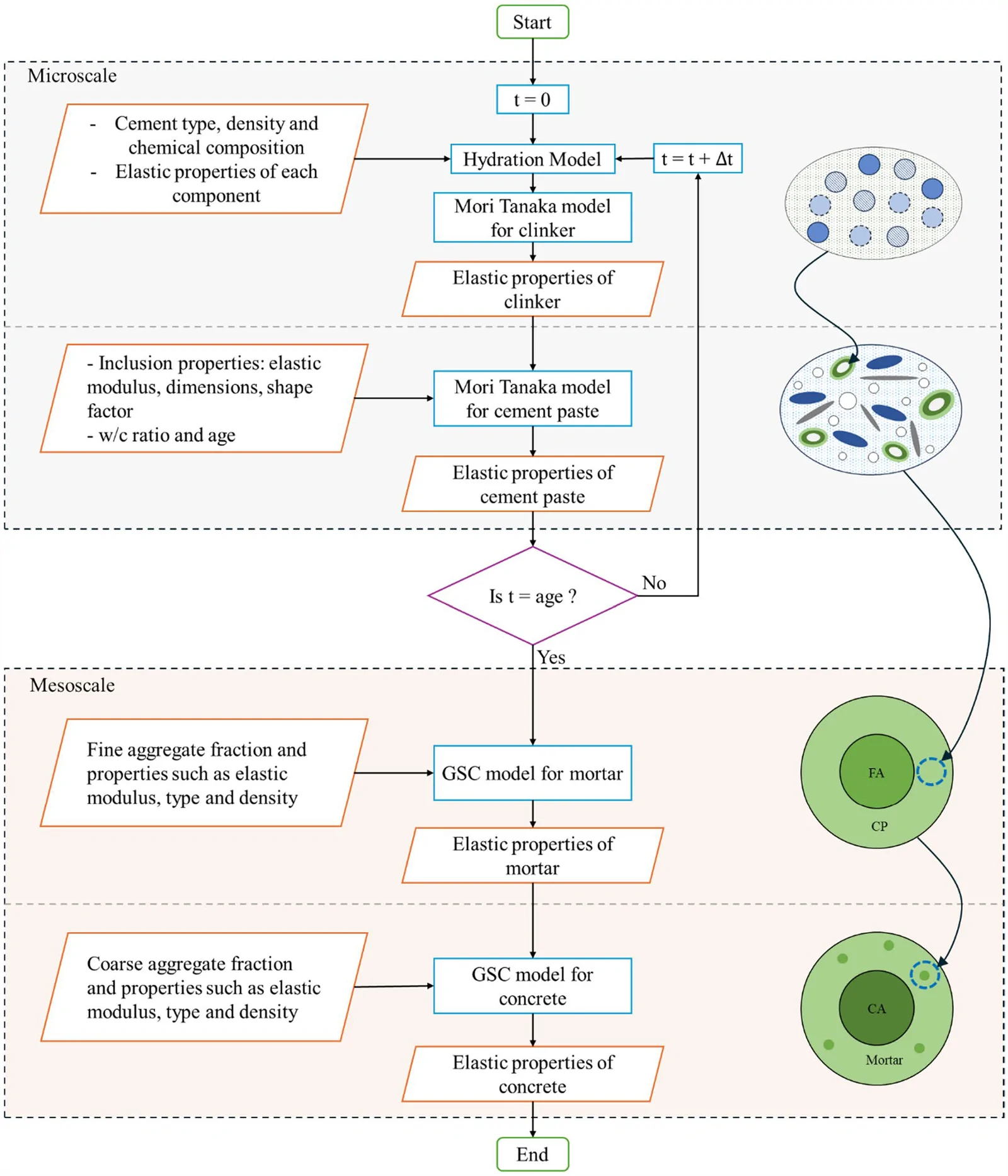
Flowchart of the proposed computational framework

**Fig. 8:**
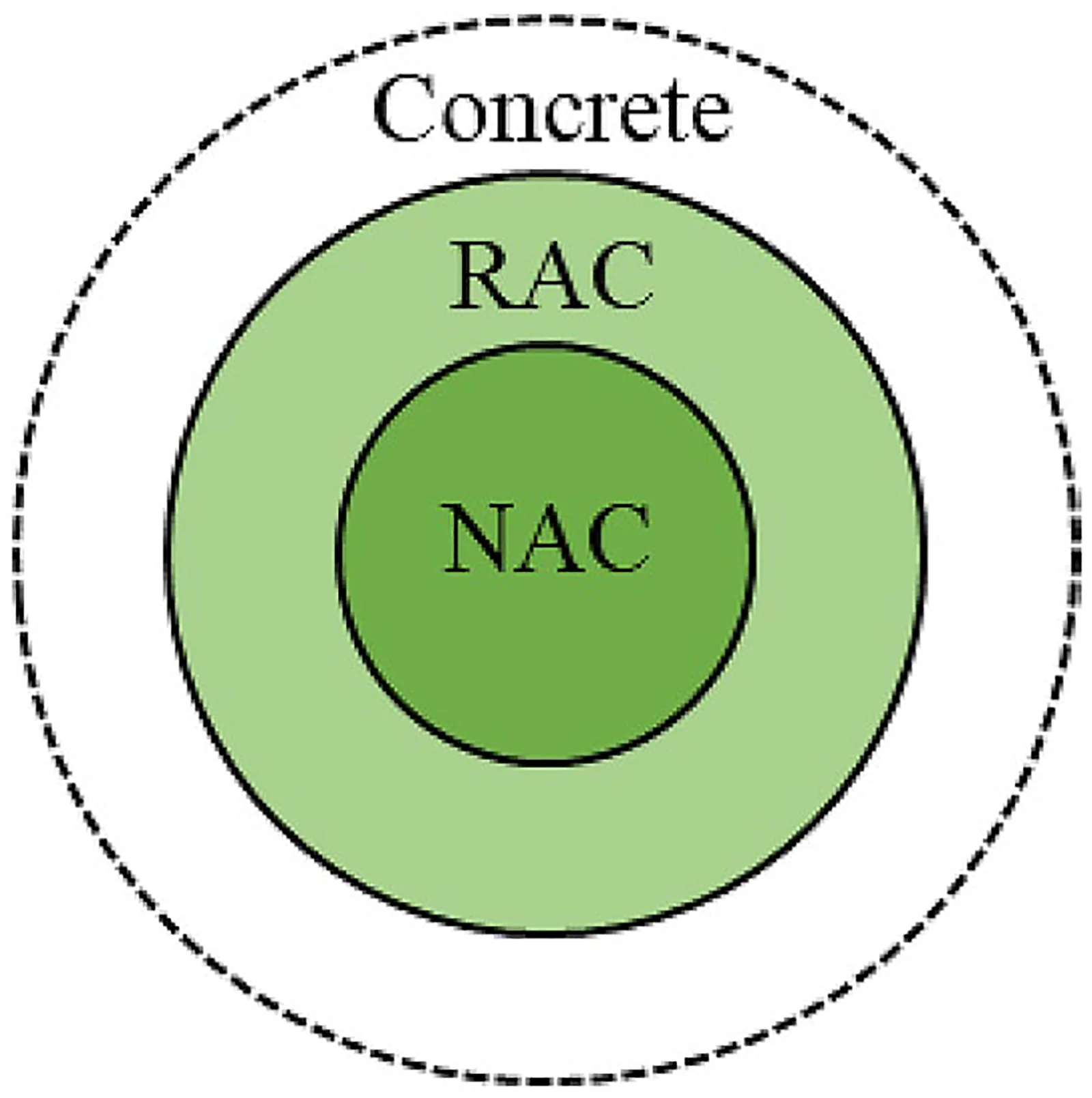
The GSC model for concrete including recycled aggregates

**Fig. 9:**
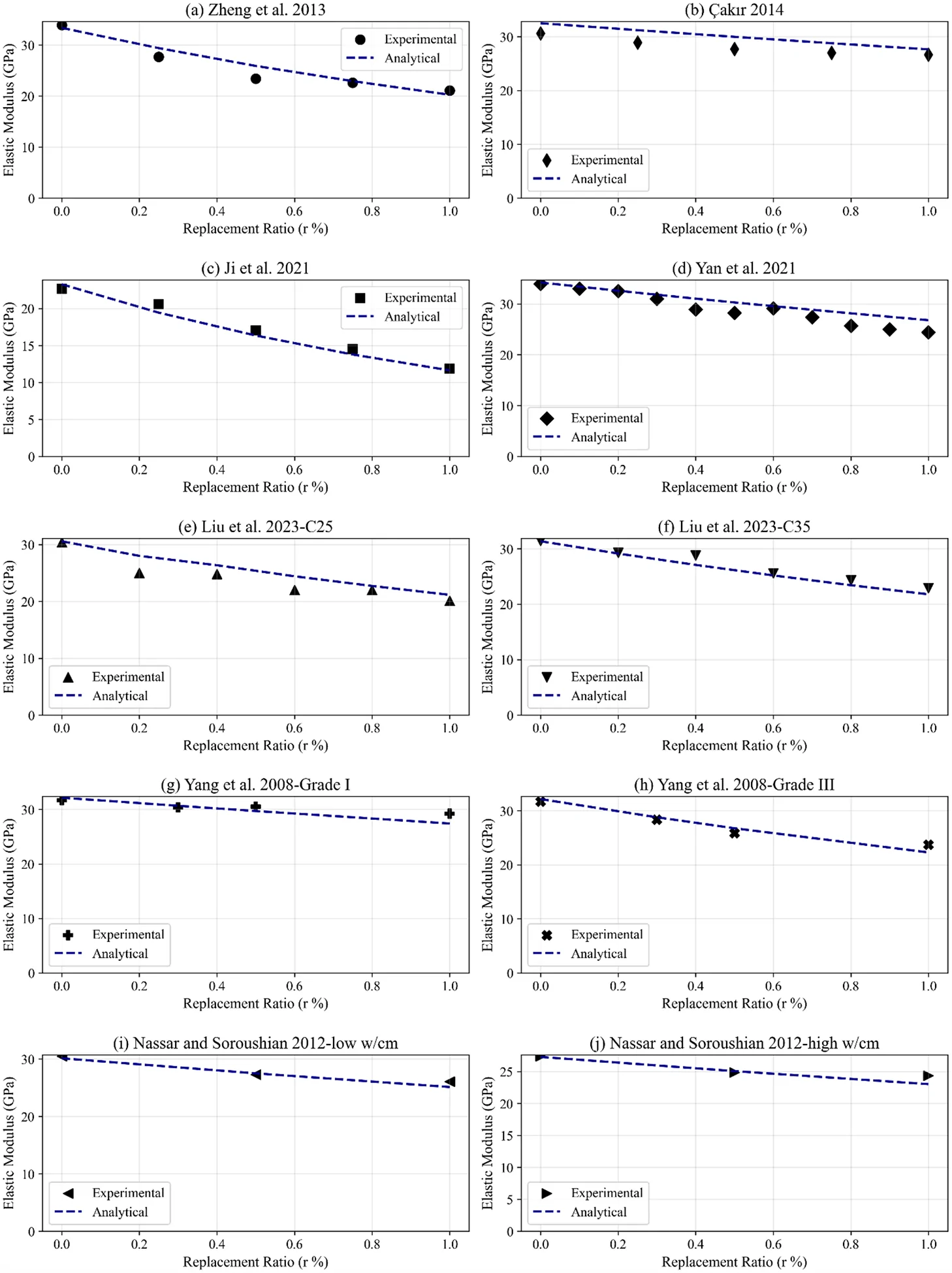
A comparison of experimental data (symbols) with analytical predictions (dashed lines): **a** Zheng et al. 2023 [32], **b** Çakır 2014 [68], **c** Ji et al. 2023 [36], **d** Yan et al. 2021 [40], **e** and **f** Liu et al. 2023 [55], **g** and **h** Yang et al. 2008 [8], **i** and **j** Nassar and Soroushian 2012 [66]

**Fig. 10:**
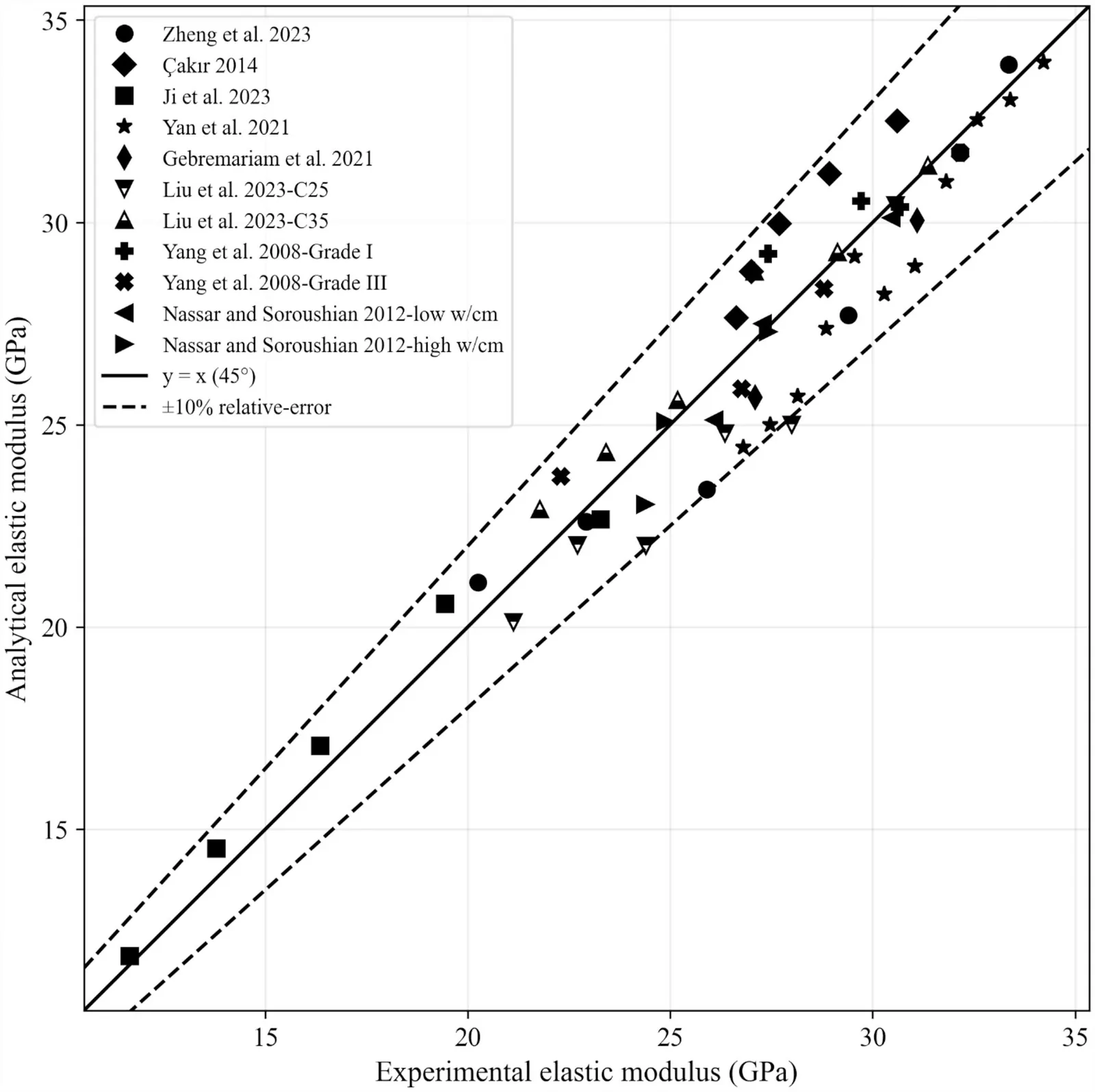
Comparison of experimental and analytical results with identity line (y=x, 45°) and ± 10% error bounds. Data from: **a** Zheng et al. 2023 [32], **b** Çakır 2014 [68], **c** Ji et al. 2023 [36], **d** Yan et al. 2021 [40], **e** Gebremariam et al. 2021 [67], **f** Liu et al. 2023 [55], **g** Yang et al. 2008 [8], **h** Nassar and Soroushian 2012 [66]

**Fig. 11:**
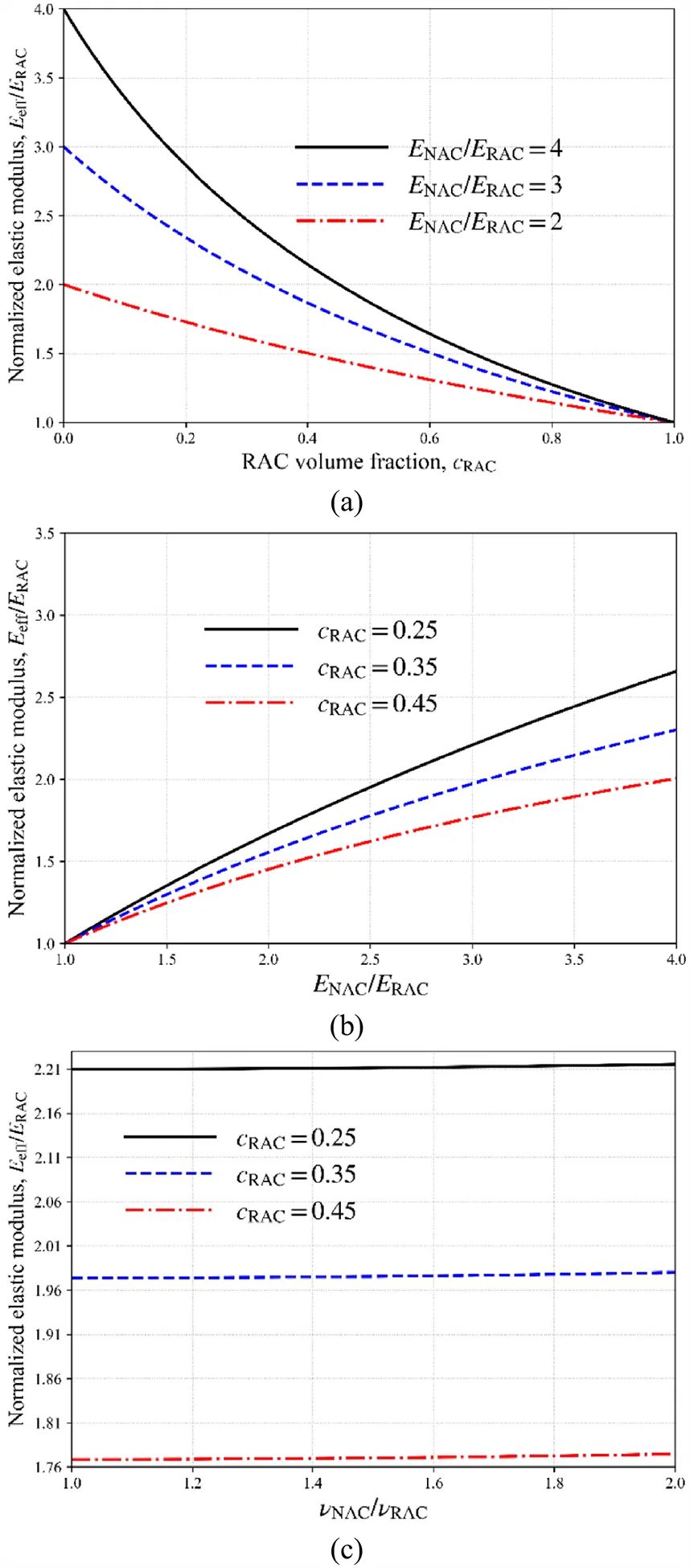
Effect on the normalized effective elastic modulus, $E_{\text{eff}}/E_{\text{RAC}}$, of **a** RAC volume fraction at different $E_{\text{NAC}}/E_{\text{RAC}}$ ratios, **b** $E_{\text{NAC}}/E_{\text{RAC}}$ at different RAC volume fractions, and **c** $v_{\text{NAC}}/v_{\text{RAC}}$ at different RAC volume fractions

**Table 1:** Effective elastic modulus of concrete based on different composite models

Model	Effective elastic modulus	
Parallel	$E_{\text{eff}}=E_{m}c_{m}+E_{i}c_{i}$	(4)
Series	$\frac{1}{E_{\text{eff}}}=\frac{c_{m}}{E_{m}}+\frac{c_{i}}{E_{i}}$	(5)
H-S	$E_{\text{eff}}=K_{i}+\frac{c_{m}}{\frac{1}{K_{m}-K_{i}}+\frac{3c_{i}}{3K_{i}+4G_{i}}}$	(6)
Counto-I	$E_{\text{eff}}=E_{m}+\frac{E_{m}c_{i}}{\sqrt{c_{i}}-c_{i}+\frac{E_{m}}{E_{i}-E_{m}}}$	(7)
Counto-II	$E_{\text{eff}}=E_{m}+\frac{E_{m}c_{i}}{\frac{E_{i}}{E_{i}-E_{m}}-\sqrt{c_{i}}}$	(8)
Hirsch	$E_{\text{eff}}=\frac{1}{2}\left(E_{m}c_{m}+E_{i}c_{i}\right)+\frac{c_{m}}{2E_{m}}+\frac{c_{i}}{2E_{i}}$	(9)
GSC	$E_{\text{eff}}=\frac{9k_{\text{eff}}G_{\text{eff}}}{3k_{\text{eff}}+G_{\text{eff}}}$	(10)

**Table 2:** Elastic modulus, Poisson’s ratios and Coefficients of the reacting compounds

Compound	E (GPa)	v	$a_{i}$	$b_{i}$	$c_{i}$
C_3_S	135	0.3	0.25	0.9	0.7
C_2_S	130	0.3	0.46	0	0.12
C_3_A	145	0.3	0.28	0.9	0.77
C_4_AF	125	0.3	0.26	0.9	0.55

**Table 3:** Mix proportion and materials properties for Stage I validation

Specimen Type	Mix proportions kg/m^3^				Density (g/cm^3^)		Poisson’s ratio		Elastic modulus (GPa)		Error (%)
	Cement	Water	FA	CA	FA	CA	FA	CA	Analytical	Exp	
Natural[8]	350	175	730	1016	2.58	2.6	0.25	0.23	32.16	31.72	1.39
RG I*[8]	350	175	730	989		2.53		0.18	27.42	29.22	6.17
RG III*^[^8]	350	175	730	938		2.4		0.18	22.29	23.72	6.00
W0[36]	487	195	613	1138	2.61	2.19	0.18	0.22	23.28	22.65	2.77
W100*[36]	487	195	613	900		2.7		0.15	11.64	11.86	1.79
Mortar[40]	461	175	512	1252	2.61	2.66	0.18	0.25	34.22	33.96	0.76
NAC[40]	461	175	512	1252		2.59		0.20	26.80	24.44	9.66
RAC-100*[40]	461	175	512	–		–		0.15	21.72	22.00	1.27
ORC0[32]	555.5	250.4	674.1	1243	2.61	2.75	0.25	0.16	33.36	33.90	1.59
ORC100*^[32]^	555.5	250.4	674.1	1243		2.27		0.24	20.25	21.10	4.05
C25 [55]	341.7	205	666.2	1237.2	2.67	2.746	0.25	0.23	30.57	30.42	0.47
C35 [55]	386.8	205	650.4	1237.2		2.491		0.18	21.13	20.12	5.02
C25*[55]	341.7	205	666.2	1207.8		2.746		0.23	31.36	31.41	0.14
C35*[55]	386.8	205	650.4	1207.8		2.491		0.18	21.77	22.91	5.01
C-Ref[67]	335	161	750	907	2.62	2.49	0.25	0.23	29.83	31.1	4.08
C-100–0*[67]	335	161	682	882		2.26		0.18	30.04	27.1	10.85
NA[68]	350	175	874	1170	2.55	2.635	0.25	0.23	25.67	30.60	16.12
RA100[68]	350	175	874	1170		2.315		0.18	27.64	26.63	3.79
M1[66]	468	178	651	952	2.65	2.57	0.25	0.23	30.11	30.46	1.15
M4*[66]	468	178	651	797		2.3		0.18	25.11	26.08	3.74
M6[66]	468	234	651	952		2.57		0.23	27.29	27.41	0.42
M9*[66]	468	234	651	797		2.3		0.18	23.03	24.38	5.51

* Indicates the samples with 100% recycled coarse aggregate (RCA)

**Table 4:** Properties of cement, chemical Composition

References	Cement Type	Chemical Composition			
		CaO	SiO2	Al2O3	Fe2O3
[8, 32, 36, 40, 55], and [66]	I	0.58	0.16	0.02	0.12
[67]	III	0.478	0.3052	0.0884	0.0114
[68]	I	0.635	0.201	0.049	0.036

## Appendix A

The coefficients for the effective shear modulus, $C_{1},\;C_{2}$, and $C_{3}$, in Eq. (3) are given as follow:

(33)
C1=8G1G2−14−5v2η1d103−263G1G2−1η2+2η1η3d73+252G1G2−1η2d53−50G1G2−17−12v2+8v22η2d+47−10v2η2η3

(34)
C2=−2G1G2−11−5v2η1d103+263G1G2−1η2+2η1η3d73−252G1G2−1η2d53+75G1G2−13−v2η2v2d+(3/2)15v2−7η2η3

(35)
C3=4G1G2−15v2−7η1d103−263G1G2−1η2+2η1η3d73+252G1G2−1η2d53+25G1G2−1v22−7η2d−7+5v2η2η3

where

(36)
η1=49−50v1v2G1G2−1+35G1G2v1−2v2+352v1−v2

(37)
η2=5v1G1G2−8+7G1G2+4

(38)
η3=G1G28−10v2+7−5v2

## Appendix B

The volume fractions of the remaining constituent phases in hardened cement paste are given as follows:

(39)
$$f_{\text{LDCSH}}=0.7f_{\text{CSH}}$$

(40)
$$f_{\text{HDCSH}}=0.3f_{CSH}-f_{\text{UHDCSH}}$$

(41)
$$\text{For}\;0.2<\frac{w}{c}\leq 0.4\;:\;f_{\text{nano}\;\text{CH}}=0.6308\;f_{\text{UHDCSH}},f_{\text{UHDCSH}}=0.2f_{CSH}$$

(42)
$$\text{For}\frac{w}{c}>0.4\;:\;f_{\text{UHDCSH}}=0,f_{\text{nanoCH}}=0$$

## References

[1] Xiong B, Demartino C, Xu J, Simi A, Marano GC, Xiao Y (2021) High-strain rate compressive behavior of concrete made with substituted coarse aggregates: Recycled crushed concrete and clay bricks. Constr Build Mater 301:123875. 10.1016/j.conbuildmat.2021.123875

[2] Xie T, Fang C (2019) Nanomaterials applied in modifications of geopolymer composites: a review. Aust J Civ Eng 17(1):32–49. 10.1080/14488353.2019.1614353

[3] Xie T, Yang G, Zhao X, Xu J, Fang C (2020) A unified model for predicting the compressive strength of recycled aggregate concrete containing supplementary cementitious materials. J Clean Prod 251:119752. 10.1016/j.jclepro.2019.119752

[4] Yadav A, Dahal D, Chhetri Sapkota S, Dumre P, Kumar Sah B (2024) Experimental study on the mechanical properties of sustainable concrete using recycled plastic and glass waste. Int J Innov Sci Res Technol 9(11):93–101

[5] Cui S (2024) Strengthening effect of nano-materials on the compressive strength and microstructure of recycled brick aggregate concrete. Medziagotyra 30(2):239–247. 10.5755/j02.ms.35298

[6] Oikonomou ND (2005) Recycled concrete aggregates. Cem Concr Compos 27(2):315–318. 10.1016/j.cemconcomp.2004.02.020

[7] Tu TY, Chen YY, Hwang CL (2006) Properties of HPC with recycled aggregates. Cem Concr Res 36(5):943–950. 10.1016/j.cemconres.2005.11.022

[8] Yang KH, Chung HS, Ashour AF (2008) Influence of type and replacement level of recycled aggregates on concrete properties. ACI Mater J 105(3):289–296. 10.14359/19826

[9] Tavakoli M, Soroushian P (1996) Strengths of recycled aggregate concrete made using field- demolished concrete as aggregate. ACI Mater J 93(2):182–190

[10] Poon CS, Kou SC, Lam L (2002) Use of recycled aggregates in molded concrete bricks and blocks. Constr Build Mater 16(5):281–289. 10.1016/S0950-0618(02)00019-3

[11] Katz A (2003) Properties of concrete made with recycled aggregate from partially hydrated old concrete. Cem Concr Res 33(5):703–711. 10.1016/S0008-8846(02)01033-5

[12] Li B (2022) Strength criterion of recycled aggregate concrete under triaxial compression: Model calibration. Constr Build Mater 320:126201. 10.1016/j.conbuildmat.2021.126201

[13] Xiao J (2018) Recycled aggregate concrete structures. Springer Nature, Berlin, Heidelberg. 10.1007/978-3-662-53987-3

[14] Sae-Long W (2025) A two-step composite mechanics approach for post-peak behavior of concrete incorporating both natural and recycled concrete aggregates. Constr Build Mater 490:142365. 10.1016/j.conbuildmat.2025.142365

[15] Dhir RK, Lye CQ (2019) Recycled Aggregates: Use in Concrete. ICE Inst Civil Eng 72(6):324

[16] Silva RV, Brito J, Dhir RK (2015) The influence of the use of recycled aggregates on the compressive strength of concrete: a review. Eur J Environ Civ Eng 19(7):825–849. 10.1080/19648189.2014.974831

[17] de Brito J, Kurda R, da Silva PR (2018) Can we truly predict the compressive strength of concrete without knowing the properties of aggregates? Appl Sci 8(7):1–21. 10.3390/app8071095

[18] Xu JJ, Chen ZP, Ozbakkaloglu T, Zhao XY, Demartino C (2018) A critical assessment of the compressive behavior of reinforced recycled aggregate concrete columns. Eng Struct 161:161–175. 10.1016/j.engstruct.2018.02.003

[19] Sae-Long W (2024) Three-phase damage model based on composite mechanics for post-peak analysis of recycled aggregate concrete. Int J Damage Mech. 10.1177/10567895241303221

[20] Wang H (2023) Effect of recycled concrete aggregate with different degrees of initial alkali–aggregate reaction damage on the mechanical behavior and porosity of self-compacting recycled aggregate concrete. Constr Build Mater 363:129797. 10.1016/j.conbuildmat.2022.129797

[21] Wardeh G, Ghorbel E, Gomart H, Fiorio B (2017) Experimental and analytical study of bond behavior between recycled aggregate concrete and steel bars using a pullout test. Struct Concr 18(5):811–825. 10.1002/suco.201600155

[22] Phiangphimai C (2023) Shrinkage, compressive and bond strengths of alkali activated/cement powder for alternative coating applications. Constr Build Mater 400:132631. 10.1016/j.conbuildmat.2023.132631

[23] Wang C, Xiao J, Qi C (2022) Dynamic damage analysis of recycled aggregate concrete structures. J Earthq Eng 26(12):6063–6081. 10.1080/13632469.2021.1911883

[24] Xiao J, Li W, Sun Z, Lange DA, Shah SP (2013) Properties of interfacial transition zones in recycled aggregate concrete tested by nanoindentation. Cem Concr Compos 37(1):276–292. 10.1016/j.cemconcomp.2013.01.006

[25] Zhou Y, Xu W, Zhuang J, Lin W, Xing F, Hu R (2024) Effects of carbonation modification on shear performance of recycled aggregate concrete beams: test and mechanism analysis. Eng Struct 315:118385. 10.1016/j.engstruct.2024.118385

[26] Li Y, Long J, Chen X (2024) Property enhancement of recycled coarse aggregate and its concrete under CO2-accelerated curing treatment. Mater. 10.3390/ma17174194 PMC11396506 39274584

[27] Jamil S, Shi J, Idrees M (2023) Effect of various parameters on carbonation treatment of recycled concrete aggregate using the design of experiment method. Constr Build Mater 382:131339. 10.1016/j.conbuildmat.2023.131339

[28] Nieto D, Dapena E, Alaejos P, Olmedo J, Pérez D (2019) Properties of self-compacting concrete prepared with coarse recycled concrete aggregates and different water:cement ratios. J Mater Civ Eng 31(2):4018376. 10.1061/(asce)mt.1943-5533.0002566

[29] Matias D, De Brito J, Rosa A, Pedro D (2013) Mechanical properties of concrete produced with recycled coarse aggregates - influence of the use of superplasticizers. Constr Build Mater 44:101–109. 10.1016/j.conbuildmat.2013.03.011

[30] Tianyu X, Aliakbar G, Togay O (2018) Toward the development of sustainable concretes with recycled concrete aggregates: comprehensive review of studies on mechanical properties. J Mater Civ Eng 30(9):4018211. 10.1061/(ASCE)MT.1943-5533.0002304

[31] Chen Z, Chen J, Ning F, Li Y (2018) Residual properties of recycled concrete after exposure to high temperatures. Mag Concr Res 71(15):781–793. 10.1680/jmacr.17.00503

[32] Zheng YQ, Zheng LY, Wang YQ (2023) Compressive performance and stress-strain relationship of geopolymer recycled brick aggregate concrete. Mag Concr Res 75(19):973–983. 10.1680/jmacr.22.00269

[33] Ge P, Huang W, Zhang J, Quan W, Guo Y (2021) Mix proportion design method of recycled brick aggregate concrete based on aggregate skeleton theory. Constr Build Mater 304:124584. 10.1016/j.conbuildmat.2021.124584

[34] Cachim PB (2009) Mechanical properties of brick aggregate concrete. Constr Build Mater 23(3):1292–1297. 10.1016/j.conbuildmat.2008.07.023

[35] Debieb F, Kenai S (2008) The use of coarse and fine crushed bricks as aggregate in concrete. Constr Build Mater 22(5):886–893. 10.1016/j.conbuild-mat.2006.12.013

[36] Ji Y, Wang D (2023) Constitutive model of waste brick concrete based on Weibull strength theory. Case Stud Constr Mater 18:e01738. 10.1016/j.cscm.2022.e01738

[37] Silva RV, De Brito J, Dhir RK (2014) Properties and composition of recycled aggregates from construction and demolition waste suitable for concrete production. Constr Build Mater 65:201–217. 10.1016/j.conbuildmat.2014.04.117

[38] Pedro D, de Brito J, Evangelista L (2017) Structural concrete with simultaneous incorporation of fine and coarse recycled concrete aggregates: Mechanical, durability and long-term properties. Constr Build Mater 154:294–309. 10.1016/j.conbuildmat.2017.07.215

[39] Younis KH, Pilakoutas K (2013) Strength prediction model and methods for improving recycled aggregate concrete. Constr Build Mater 49:688–701. 10.1016/j.conbuildmat.2013.09.003

[40] Yan S, Wang B, Sun Y, Lyu B (2021) Micromechanics-based prediction models and experimental validation on elastic modulus of recycled aggregate concrete. Sustainability. 10.3390/su132011172

[41] Li Z, Li LY, Cheng S (2024) Evaluation of modulus of elasticity of concrete containing both natural and recycled concrete aggregates. J Clean Prod 447:14591. 10.1016/j.jclepro.2024.141591

[42] Dehwah OHA, Xi Y (2025) Theoretical modeling of the effective thermal conductivity of concrete. J Mater Civ Eng 37(11):4025396. 10.1061/JMCEE7.MTENG-19677

[43] Christensen RM (1979) Mechanics of composite materials. Wiley Interscience, New York, NY, USA

[44] Christensen RM (2008) Mechanics of composite materials. Mech Compos Mater 44(3):197–198. 10.1007/s11029-008-9011-3

[45] Zhou FP, Lydon FD, Barr BIG (1995) Effect of coarse aggregate on elastic modulus and compressive strength of high performance concrete. Cem Concr Res 25(1):177–186. 10.1016/0008-8846(94)00125-I

[46] Torquato S (2002) Random heterogeneous materials. Springer, New York. 10.1007/978-1-4757-6355-3

[47] Lee JK (2007) Prediction of thermal conductivity of composites with spherical fillers by successive embedding. Arch Appl Mech 77(7):453–460. 10.1007/s00419-006-0108-7

[48] Carson JK, Lovatt SJ, Tanner DJ, Cleland AC (2005) Thermal conductivity bounds for isotropic, porous materials. Int J Heat Mass Transf 48(11):2150–2158. 10.1016/j.ijheatmasstransfer.2004.12.032

[49] Dehwah OHA (2024), “Modeling of Electrokinetic Nanoparticle Injection and Coupling Effects in Hygro-Thermo-Chemical Transport in Concrete,” University of Colorado Boulder. Available: https://research.ebsco.com/linkprocessor/plink?id=a3a73bd8-aa43-3a27-8e13-36c815cddb3e

[50] Hashin Z, Shtrikman S (1962) A variational approach to the theory of the elastic behaviour of polycrystals. J Mech Phys Solids 10(4):343–352. 10.1016/0022-5096(62)90005-4

[51] Hashin Z, Shtrikman S (1963) A variational approach to the theory of the elastic behaviour of multiphase materials. J Mech Phys Solids 11(2):127–140. 10.1016/0022-5096(63)90060

[52] Hashin Z, Shtrikman S (1962) A variational approach to the theory of the effective magnetic permeability of multiphase materials. J Appl Phys 33(10):3125–3131. 10.1063/1.1728579

[53] Counto UJ (1964) The effect of the elastic modulus of the aggregate on the elastic modulus, creep and creep recovery of concrete. Mag Concr Res 16(48):129–138. 10.1680/macr.1964.16.48.129

[54] Hirsch TJ (1962) Modulus of Elasticity iof Concrete Affected by Elastic Moduli of Cement Paste Matrix and Aggregate. ACI J Proc. 59(3):427–452

[55] Liu B, Zhang B, Wang ZZ, Bai GL (2023) Study on the stress-strain full curve of recycled coarse aggregate concrete under uniaxial compression. Constr Build Mater 363:129884. 10.1016/j.conbuildmat.2022.129884

[56] Mori T, Tanaka K (1973) Average stress in matrix and average elastic energy of materials with misfitting inclusions. Acta Metall 21(5):571–574. 10.1016/0001-6160(73)90064-3

[57] Alsharari BS, Dehwah OHA, Xi Y (2025) Modeling the degradation of aggregate properties under neutron radiation. Constr Build Mater 489:142391. 10.1016/j.conbuildmat.2025.142391

[58] Vandamme M, Ulm FJ, Fonollosa P (2010) Nanogranular packing of C-S-H at substochiometric conditions. Cem Concr Res 40(1):14–26. 10.1016/j.cemconres.2009.09.017

[59] Chen J, Jin X, Jin N, Tian Y (2014) A nano-model for micromechanics-based elasticity prediction of hardened cement paste. Mag Concr Res 66(22):1145–1153. 10.1680/macr.14.00084

[60] Stora E, He QC, Bary B (2006) Influence of inclusion shapes on the effective linear elastic properties of hardened cement pastes. Cem Concr Res 36(7):1330–1344. 10.1016/j.cemconres.2006.02.007

[61] Jing Y, Xi Y (2025) Modeling the mechanical behavior and damage development of concrete materials under neutron irradiation. Front Struct Civ Eng 19(9):1545–1562

[62] Damien D, Wang Y, Xi Y (2019) Prediction of elastic properties of cementitious materials based on multiphase and multiscale micromechanics theory. J Eng Mech. 10.1061/(asce)em.1943-7889.0001650

[63] Termkhajornkit P, Vu QH, Barbarulo R, Daronnat S, Chanvillard G (2014) Dependence of compressive strength on phase assemblage in cement pastes: beyond gel-space ratio - experimental evidence and micromechanical modeling. Cem Concr Res 56:1–11. 10.1016/j.cemconres.2013.10.007

[64] Xi Y, Jennings HM (1997) Shrinkage of cement paste and concrete modelled by a multiscale effective homogeneous theory. Mater Struct Constr 30(6):329–339. 10.1007/bf02480683

[65] Taylor HFW (1987) A method for predicting alkazi ion concentrations in cement pore solutions. Adv Cem Res 1(1):5–17. 10.1680/adcr.1987.1.1.5

[66] Nassar RUD, Soroushian P (2012) Strength and durability of recycled aggregate concrete containing milled glass as partial replacement for cement. Constr Build Mater 29:368–377. 10.1016/j.conbuildmat.2011.10.061

[67] Gebremariam AT (2021) Comprehensive study on the most sustainable concrete design made of recycled concrete, glass and mineral wool from C&D wastes. Constr Build Mater 273:121697. 10.1016/j.conbuildmat.2020.121697

[68] Çakir O (2014) Experimental analysis of properties of recycled coarse aggregate (RCA) concrete with mineral additives. Constr Build Mater 68:17–25. 10.1016/j.conbuildmat.2014.06.032

